# Digital Adherence Technologies Linked to Mobile Money Incentives for Medication Adherence Among People Living With Tuberculosis: Mixed Methods Feasibility and Acceptability Study

**DOI:** 10.2196/47996

**Published:** 2024-05-31

**Authors:** Angella Musiimenta, Wilson Tumuhimbise, Esther Atukunda, Aaron Mugaba, Sebastian Linnemayr, Jessica Haberer

**Affiliations:** 1 Faculty of Computing and Informatics Mbarara University of Science and Technology Mbarara Uganda; 2 Angels Compassion Research and Development Initiative Mbarara Uganda; 3 Faculty of Medicine Mbarara University of Science and Technology Mbarara Uganda; 4 Rand Corporation Santa Monica, CA United States; 5 Harvard Medical School Boston, MA United States; 6 Massachusetts General Hospital Center for Global Health Boston, MA United States

**Keywords:** digital adherence technologies, real-time monitoring, SMS text message reminders, mobile money, financial incentives, tuberculosis, medication adherence, user-centered approach

## Abstract

**Background:**

Complementing digital adherence technologies (DATs) with mobile money incentives may improve their utility in supporting tuberculosis medication adherence, yet the feasibility and acceptability of this integrated approach remain unclear.

**Objective:**

This study aims to describe the feasibility and acceptability of a novel DAT intervention called My Mobile Wallet composed of real-time adherence monitoring, SMS text message reminders, and mobile money incentives for tuberculosis medication adherence in a low-income setting.

**Methods:**

We purposively recruited people living with tuberculosis from the Mbarara Regional Referral Hospital in Mbarara, Uganda, who (1) were starting tuberculosis treatment at enrollment or within the past 4 weeks, (2) owned a mobile phone, (3) were able to use SMS test messaging, (4) were aged ≥18 years, and (5) were living in Mbarara district. At study exit (month 6), we used interviews and questionnaires informed by the unified theory of acceptance and use of technology (UTAUT) to collect feasibility and acceptability data, reflecting patients’ experiences of using each component of My Mobile Wallet. Feasibility also included tracking the functionality of the adherence monitor (ie, an electronic pillbox) as well as SMS text message and mobile money delivery. We used a content analytical approach to inductively analyze qualitative data and Stata (version 13; StataCorp LLC) to analyze quantitative data.

**Results:**

All 39 participants reported that the intervention was feasible because it was easy for them to use (eg, access and read SMS text messages) and worked as expected. Almost all SMS text messages (6880/7064, 97.4%) were sent as planned. The transmission of adherence data from the monitor worked well, with 98.37% (5682/5776) of the data transmitted as planned. All participants additionally reported that the intervention was acceptable because it helped them take their tuberculosis medication as prescribed; the mobile money incentives relieved them of tuberculosis-related financial burdens; SMS text message reminders and electronic pillbox–based alarms reminded them to take their medication on time; and participants perceived real-time adherence monitoring as “being watched” while taking their medication, which encouraged them to take their medication on time to demonstrate their commitment. The intervention was perceived as a sign of care, which eventually created emotional support and a sense of connectedness to health care. Participants preferred daily SMS text message reminders (32/39, 82%) to reminders linked to missed doses (7/39, 18%), citing the fact that tuberculosis medication is taken daily.

**Conclusions:**

The use of real-time adherence monitoring linked to SMS text message reminders and mobile money incentives for tuberculosis medication adherence was feasible and acceptable in a low-resource setting where poverty-based structural barriers heavily constrain tuberculosis treatment and care.

## Introduction

### Background

Tuberculosis treatment adherence remains challenging in Uganda. Constraints to tuberculosis medication adherence include a lack of transport to the clinic to pick up the drugs and forgetfulness [1]. Digital adherence technologies (DATs) are being explored to encourage adherence to tuberculosis medication [2,3]. Recently, we showed that real-time adherence monitors linked to SMS text message reminders were potentially useful in reminding patients to take their medication and encouraging tuberculosis medication adherence in rural Uganda [4]. However, tuberculosis is well known to be a disease of poverty [5], and the lack of money may potentially limit the usefulness of DATs (eg, the inability to afford transport to pick up the medications) [6]. Although effective tuberculosis treatment has existed since the 1940s and is available for free, many people delay seeking treatment, struggle with medication adherence, or do not complete their treatment because of poverty [1]. This is because tuberculosis leads to the loss of productivity of patients and their caregivers, resulting in additional costs for patients in the form of transport to and from the clinic and may lead to loss of employment for fear of spreading the disease to other people [7]. Currently, in Uganda, 53% of patients living with tuberculosis take loans or sell property to meet the costs of their tuberculosis care [8]. Interventions are necessary to overcome the poverty-based structural barriers to tuberculosis treatment, including unconditional transport to and from the clinic. According to the End TB strategy of the World Health Organization (WHO), the use of social protection schemes (such as transport to the clinic and meals) could lower the financial burden of tuberculosis [9]. A recent systematic literature review and meta-analysis by Richterman et al [10] defines cash transfers as cash payments provided to specific beneficiaries. The review indicates that cash transfer interventions may improve treatment success among patients with pulmonary tuberculosis, although the review expresses the need for more research regarding the effectiveness of sensitive cash transfers for tuberculosis care, especially in low-income countries [10].

The use of mobile money technology (money sent, received, or saved on mobile phones) is a promising tool for delivering health-related cash transfers; for instance, mobile money enabled pregnant women to save for maternal health care in Kenya [11], while a progressive incentive scheme to reward private physicians and community health care workers enhanced identification and referral of suspected tuberculosis cases and treatment tracking in Pakistan [12]. The use of mobile money transfers to incentivize patients living with tuberculosis to take their drugs may potentially improve their adherence to medication [13]. However, the use of mobile money services in the context of health care is still in its infancy, and the limited research in this area reports mixed results [14].

### My Mobile Wallet

My Mobile Wallet is a DAT intervention composed of a real-time adherence monitor, SMS text message reminders, and mobile money incentives (known as WiseCash). The financial incentives are meant to motivate participants to take their medication as well as enable them to attend their clinic appointments for pill refills. The intervention was developed through user-centered approaches [15], and we previously published formative qualitative findings indicating the anticipated benefits and challenges of using the intervention for tuberculosis medication adherence in rural Uganda [13]. In brief, participants reported that the intervention could remind them to take their medication as well as support, and motivate tuberculosis medication adherence. However, they expressed concerns about the possible unintended tuberculosis status disclosure as well as the possibility of using the money for other competing demands. This information was then used to refine and improve My Mobile Wallet.

This paper presents the feasibility and acceptability of a pilot study implementing My Mobile Wallet. Specifically, we present the practical experiences of people living with drug-sensitive tuberculosis who used the intervention during their 6-month tuberculosis treatment period.

## Methods

### Study Design and Setting

This study used a convergent mixed methods study design. The study recruited people living with tuberculosis from the tuberculosis clinic at the Mbarara Regional Referral Hospital (MRRH) in Mbarara in southwestern Uganda. The tuberculosis clinic provides care to an estimated 600 people living with tuberculosis annually. All newly diagnosed people living with tuberculosis receive free tuberculosis medication and are counseled about the benefits of tuberculosis medication at the tuberculosis clinic. At the MRRH, the recommended directly observed therapy approach (which advises that patients should take their medication as they are watched by a health care provider or treatment supporter) is not used for monitoring medication adherence due to the costs involved for both people living with tuberculosis and the health care workers. Instead, people living with tuberculosis are treated with the 2HRZE regimen (isoniazid, rifampin, pyrazinamide, and ethambutol for 2 months) in the initiation phase, with clinic visits every 2 weeks. At the end of the 2-month period, they return to the tuberculosis clinic for a sputum conversion check. Those who become smear negative continue with the 4HR regimen (isoniazid plus rifampin for 4 months) in the continuation phase, with monthly clinic visits. Those with positive test results receive GeneXpert to exclude rifampicin resistance; subsequent treatment is then individualized. Treatment may be extended up to a full year to compensate for missed medication pick-ups or doses.

### Selection of Study Participants

**Figure 1:**
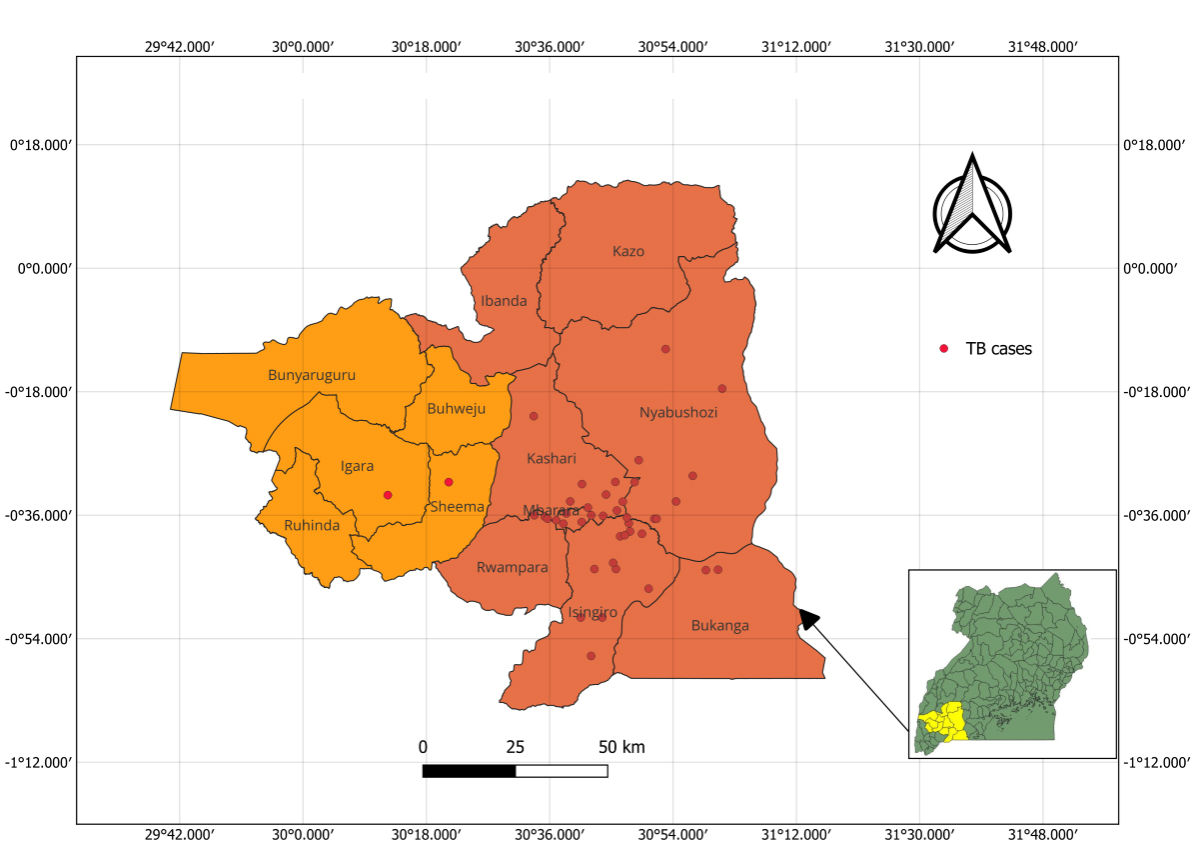
The study area map and geographic distribution of participants. TB: tuberculosis.

Between July 2022 and October 2022, we recruited participants at the MRRH according to the following inclusion criteria: (1) newly diagnosed with tuberculosis per the clinic records and starting tuberculosis treatment at enrollment or within the past 4 weeks, (2) owning a mobile phone, and (3) living in Mbarara district (Figure 1). We excluded individuals who were unwilling or unable to provide informed consent due to severe mental conditions per the clinical records and those unable to use mobile money–based SMS text messaging (we trained potential participants and tested this skill at recruitment). We purposively sampled patients to achieve relatively balanced representation by HIV status and sex to solicit diverse perspectives.

### Intervention Technology

**Figure 2:**
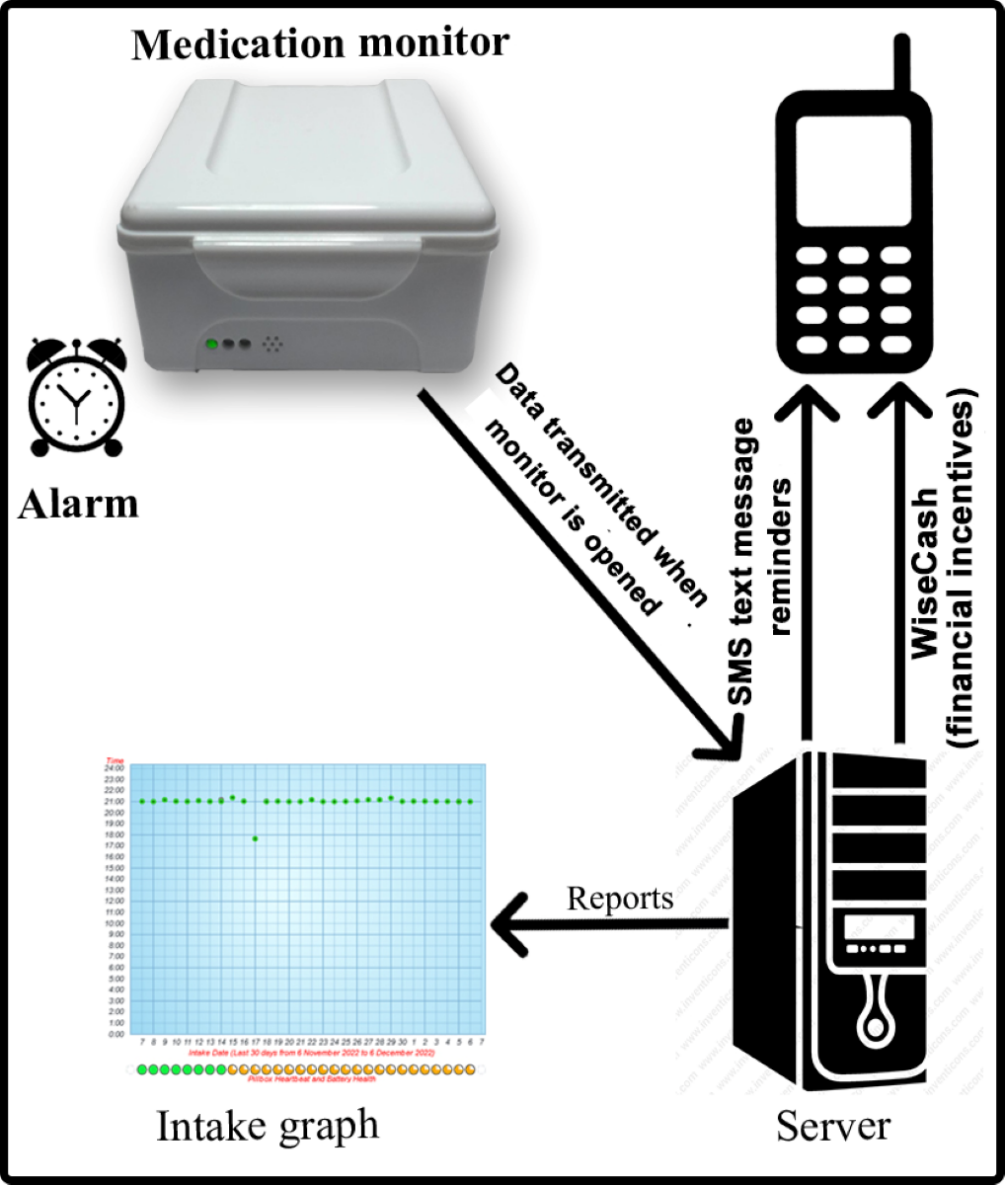
The My Mobile Wallet intervention diagram.

Details of the My Mobile Wallet intervention are described elsewhere [13]. Briefly, as shown in Figure 2, the intervention is composed of the following 3 components: a real-time medication monitor (Wisepill evriMED1000) to monitor medication adherence by sending signals when opened (the monitor records a date-and-time stamp as a proxy for taking medication, and it has an option to set an alarm sound to remind patients to take their medication); SMS text message reminders sent to users’ mobile phones to remind them to take their medication as prescribed (reminders are sent daily for 2 months, after which they are triggered as needed by missed or delayed doses); and the WiseCash app, which uses a tailored mobile money platform for sending financial incentives for transport to the clinic and motivating medication adherence.

### Study Procedures

We first oriented each participant to the My Mobile Wallet intervention components. We explained and demonstrated how the real-time adherence monitor (Wisepill) works, including how it monitors medication adherence and sends a signal to researchers every time it is opened, how the monitor makes an alarm sound to remind patients to take their medication, and how to open and close the monitor to put in or retrieve medication. Participants were then asked to demonstrate how the monitor works. Next, we explained to them how the intervention sends daily SMS text message reminders (30 minutes before medication-taking time) to remind participants to take their medication for the first 2 months. We also explained how the intervention sends SMS text message reminders for the next 4 months only if the monitor is not opened within an hour of the expected time (known as triggered SMS text message reminders). We then explained how the intervention transfers USh 28,000 (approximately equivalent to US $8; we decided upon this amount based on the transport costs, which had increased during the COVID-19 pandemic and had not reduced at the time of implementing this intervention) as an unconditional monthly mobile money incentive to mobile phones belonging to people living with tuberculosis for facilitating transport to the clinic (Figure 1 shows a visual representation of the geographic distribution of the participants) from the date of study recruitment until the end of their 6-month treatment period; furthermore, the participants were informed that USh 5250 (approximately equivalent to US $1.50) would be transferred as a monthly conditional medication adherence incentive to those with a medication adherence rate of ≥90% as ascertained from the real-time adherence monitor. The transfer of the transport incentives required patients to inform the research staff about their next date of appointment so that it could be input into the WiseCash application to allow automatic triggering of the transfer of the transport incentive a day before their next visit. We decided upon the medication adherence rate of >90% because evidence shows that adherence below this level does not yield favorable treatment outcomes [16].

### Data Collection

We administered a baseline demographic and sociobehavioral questionnaire to participants at enrollment, which included age, sex, tuberculosis medication specifications (drugs and planned dosing times), and mobile phone number and use. We used the interviewer-administered approach for administering questionnaires to elicit quantitative data orally from the participants (ie, closed-ended questions read out in the participants’ local language by the researcher, with participants answering the questions orally). Several validated surveys were adopted and included in this questionnaire (eg, the Duke-UNC Functional Social Support Questionnaire for measuring social support [17], the asset index scale to assess socioeconomic status [18], the depression section of the Hopkins Symptom Checklist for assessing depression [19], the Household Food Insecurity Access Scale for measuring food insecurity [20], the Alcohol Use Disorders Identification Test for assessing alcohol consumption [21], and the Internalized AIDS-Related Stigma Scale for assessing stigma [22]). Feasibility was ascertained by tracking the functionality of the monitor and SMS text message and mobile money delivery. The unified theory of acceptance and use of technology (UTAUT) model [23], given its track record of predicting a substantial portion of the acceptance of digital health interventions, provided a basis for developing surveys and interview guides for capturing participants’ views on feasibility and acceptability at study exit (month 6). The UTAUT model asserts that the adoption of technology is influenced by four major constructs as perceived by an individual user: (1) performance expectancy or perceived usefulness of the intervention (in this case, My Mobile Wallet) (2) effort expectancy or perceived ease of use of the intervention, (3) social norms (how others perceive the individual’s use of the intervention), and (4) facilitating conditions (the availability of technical and organizational infrastructure to support the use of the intervention). A structured exit questionnaire aimed at eliciting closed-ended information from participants regarding their experiences of using My Mobile Wallet was administered. This was a Likert scale questionnaire that sought to explore the extent to which participants liked or disliked the functionalities of the intervention. Qualitative open-ended interviews, by contrast, elicited in-depth information about participants’ experiences using each component of My Mobile Wallet (the real-time monitor, SMS text message reminders and monitor alarms, and the WiseCash application), including benefits and challenges related to the technologies. Authors WT and ATM (who are trained in qualitative research and research ethics) conducted the semistructured in-depth interviews with participants in a private space at a research office near the MRRH until thematic saturation was reached at the 30th participant interview. Each interview lasted between 30 and 60 minutes and was conducted in the local language (Runyankole), digitally recorded, transcribed, and translated into English. After each interview, author AM reviewed the transcripts for quality, clarity, and detail.

### Data Analysis

We followed the UTAUT model [23] to review transcripts for content related to acceptability. We then developed a coding scheme, used it to code the data, and reviewed the coded data to develop descriptive categories. We mapped the descriptive categories onto the domains of the UTAUT model’s four major constructs that influence technology adoption: (1) performance expectancy or perceived usefulness, (2) effort expectancy or perceived ease of use, (3) social norms, and (4) facilitating conditions. Illustrative quotations were then selected from the coded data. After the completion of the codebook, we applied the codes using NVivo 11 (Lumivero). We followed the COREQ (Consolidated Criteria for Reporting Qualitative Research) [24] checklist in reporting qualitative results. Feasibility metrics and the quantitative assessment of acceptability were analyzed descriptively by WT and ATM using Stata 13.

### Ethical Considerations

The institutional review committees of Mbarara University of Science & Technology (MUST-2021-102) and the Uganda National Council for Science and Technology (HS1688ES) approved this study. All participants provided signed informed consent before study participation.

## Results

### Demographic Characteristics

Of the 54 screened participants, we excluded 5 (9%) for not owning a mobile phone, 5 (9%) for not living within 60 kilometers of Mbarara district, and 4 (7%) for having mobile phone numbers that were not registered for mobile money service. Thus, 40 (74%) of the 54 screened participants were enrolled in the study and used the intervention for 6 months. Of these 40 participants, 1 (2%) was lost to follow-up (her mobile phone was unreachable). As indicated in Table 1, of the 40 participants, 24 (60%) were female, 27 (68%) had coinfection with HIV, 34 (85%) had no regular or fixed income, 18 (45%) did not study beyond primary level (typically attended by children aged 6-12 years), 40 (100%) perceived their social support to be insufficient, and 21 (53%) reported severe food insecurity. The participants’ median age was 38 (IQR 28-54) years, and, before joining the study, they were on medication for a median of 4 (IQR 2.5-8) weeks.

**Table 1:** Baseline demographic characteristics of participants (n=40).

Characteristics		Values
Age (y), median (IQR)		38 (28-54)
Weeks on medication before joining the study, median (IQR)		4 (2.5-8)
**Sex, n (%)**		
	Male	24 (60)
	Female	16 (40)
**Education, n (%)**		
	None	3 (8)
	P1-P7^a^	18 (45)
	Ordinary level	9 (22)
	Advanced level	3 (8)
	Tertiary level	7 (18)
**Income (fixed wages or salary), n (%)**		
	Yes	6 (15)
	No	34 (85)
**Heavy alcohol consumption, n (%)**		
	Yes	1 (2)
	No	39 (98)
Enough social support (no), n (%)		40 (100)
**Food insecurity, n (%)**		
	Yes	21 (52)
	No	19 (48)
**Probable depression, n (%)**		
	Yes	1 (2)
	No	39 (98)
**Asset index scale^b^, n (%)**		
	Lowest quartile	16 (40)
	25%-100% quartiles	24 (60)
**HIV status, n (%)**		
	Negative	13 (32)
	Positive	27 (68)

^a^In the Ugandan education system, primary school (P1-P7) is often attended by children aged 6 to 12 years.

^b^Index was measured using the measure proposed by Filmer and Pritchett [18].

As indicated in Table 2, at baseline, half of the participants (20/40, 50%) did not share their mobile phones with anyone, 85% (34/40) checked their SMS text messages more frequently than *often* in a week, 75% (30/40) *often* used mobile money, 88% (35/40) preferred receiving SMS text message reminders daily because medication taking is a daily activity, 68% (27/40) preferred SMS text message reminders that are not easily related to tuberculosis (eg, “Hello today”) to avoid unwanted tuberculosis status disclosure, and 38% (15/40) preferred receiving mobile money incentives for transport to the clinic a day before the clinic visit to avoid using the money for other competing needs.

**Table 2:** Mobile phone use and intervention preferences at baseline (n=40).

Questions		Participants, n (%)
**Who else uses your mobile phone?**		
	Spouse	3 (8)
	Family member	16 (40)
	Neighbor	1 (2)
	No one else	20 (50)
**Check for SMS text messages in a week**		
	Never	1 (2)
	Less than *often*	5 (12)
	More than *often*	34 (85)
**Use of mobile money**		
	Less than *often*	10 (25)
	More than *often*	30 (75)
**Reasons for delay in checking for SMS text messages last week^a^**		
	Mobile phone not charged	20 (50)
	Mobile phone was used by someone else	2 (5)
	No adequate signal	9 (22)
	Mobile phone not functioning	1 (2)
	Used by someone else	2 (5)
**Preferred frequency of receiving SMS text message reminders**		
	Daily	35 (88)
	Weekly	5 (12)
**Preferred content for SMS text message reminders**		
	Not easily related to TB^b^ (eg “Hello today”)	27 (68)
	Easily related to TB (eg, “Take your TB drugs”)	13 (32)
**SMS text message language preference**		
	Local language	22 (55)
	English	18 (45)
**When to send the mobile money incentive for transport to the clinic**		
	1 day before the clinic visit	25 (63)
	2 days before the clinic visit	10 (25)
	>2 days before the clinic visit	5 (12)

^a^Reasons for delay in checking for SMS text messages last week, n=21, 52%.

^b^TB: tuberculosis.

### Exit Survey Results

All participants self-reported that it was easy for them to access and read the mobile money SMS text messages as well as the medication-taking reminders (39/39, 100%) and open the Wisepill device to retrieve their medication (39/39, 100%). In addition, all participants (39/39, 100%) received the mobile money incentive for transport to the clinic as expected, received the medication adherence incentives as expected, and reported that the real-time adherence monitor worked as expected. All participants (39/39, 100%) additionally reported that the mobile money incentives, the Wisepill device, and the SMS text message reminders helped them take their tuberculosis medication on time or as prescribed.

The average adherence rate ascertained from the real-time monitors was 90.4% (SD 8.6%), and 24 (60%) of the 40 participants had an adherence rate of >90%.

As indicated in Table 3, almost all participants (38/40, 95%) opted to be reminded by both SMS text message reminders and alarms from the real-time monitor. Of the 40 participants, 2 (5%) requested study staff to switch off the alarms on their monitors at enrollment because they anticipated being inconvenienced by the sound. Participants preferred daily SMS text message reminders (32/39, 82%) to reminders linked to missed doses (7/39, 18%), citing the fact that tuberculosis medication is taken daily. All participants reported that the mobile money incentives were sent as expected. However, 4 (10%) of the 40 participants received cash (once during the study period) as refund for the transport fare instead of being sent the mobile money incentive for transport to the clinic. These participants did not inform the study staff on time about their next clinic visit date, which had to be input into the WiseCash application to allow automatic triggering of the transfer of the incentive a day before the clinic visit. Almost all the SMS text messages (6880/7064, 97.4%) were sent as planned. The transmission of adherence data from the monitor worked well, with 98.37% (5682/5776) of the data transmitted as planned. No real-time adherence monitor malfunctioned during the study period.

**Table 3:** Feasibility and acceptability of the My Mobile Wallet intervention.

Feasibility and acceptability of SMS text messages			Values
**Preference of SMS text message reminders versus device-based alarms (recorded at recruitment; n=40), n (%)**			
	Participants who opted to be reminded by SMS text message reminders only	0 (0)	
	Participants who opted to be reminded by Wisepill device alarm only	2 (5)	
	Participants who opted for SMS text message reminders plus Wisepill device alarm	38 (95)	
**SMS text message reminders (automatically ascertained from the intervention; N=7064), n (%)**			
	Total number of SMS text message reminders sent	6880 (97.4)	
	SMS text message reminders not sent due to technical challenges (eg, poor network)	184 (2.6)	
**Mobile money incentives (automatically ascertained from the intervention; N=40), n (%)**			
	Transport incentives not sent	0 (0)	
	Transport incentives sent unnecessarily	0 (0)	
	Adherence incentives not sent	0 (0)	
	Adherence incentives sent unnecessarily	0 (0)	
**Feasibility and acceptability of the real-time adherence monitor, n (%)**			
	Data loss due to technical issues with the real-time monitors (days when the monitor was not opened; automatically ascertained from the intervention; N=5776)	94 (1.63)	
	Device malfunction (N=40; devices that malfunctioned and were replaced)	0 (0)	
	Devices successfully returned by participants (N=40; the device used by the participant who was lost to follow-up was later recovered from her treatment supporter)	40 (100)	

### Interview Results: Intervention Acceptability

**Figure 3:**
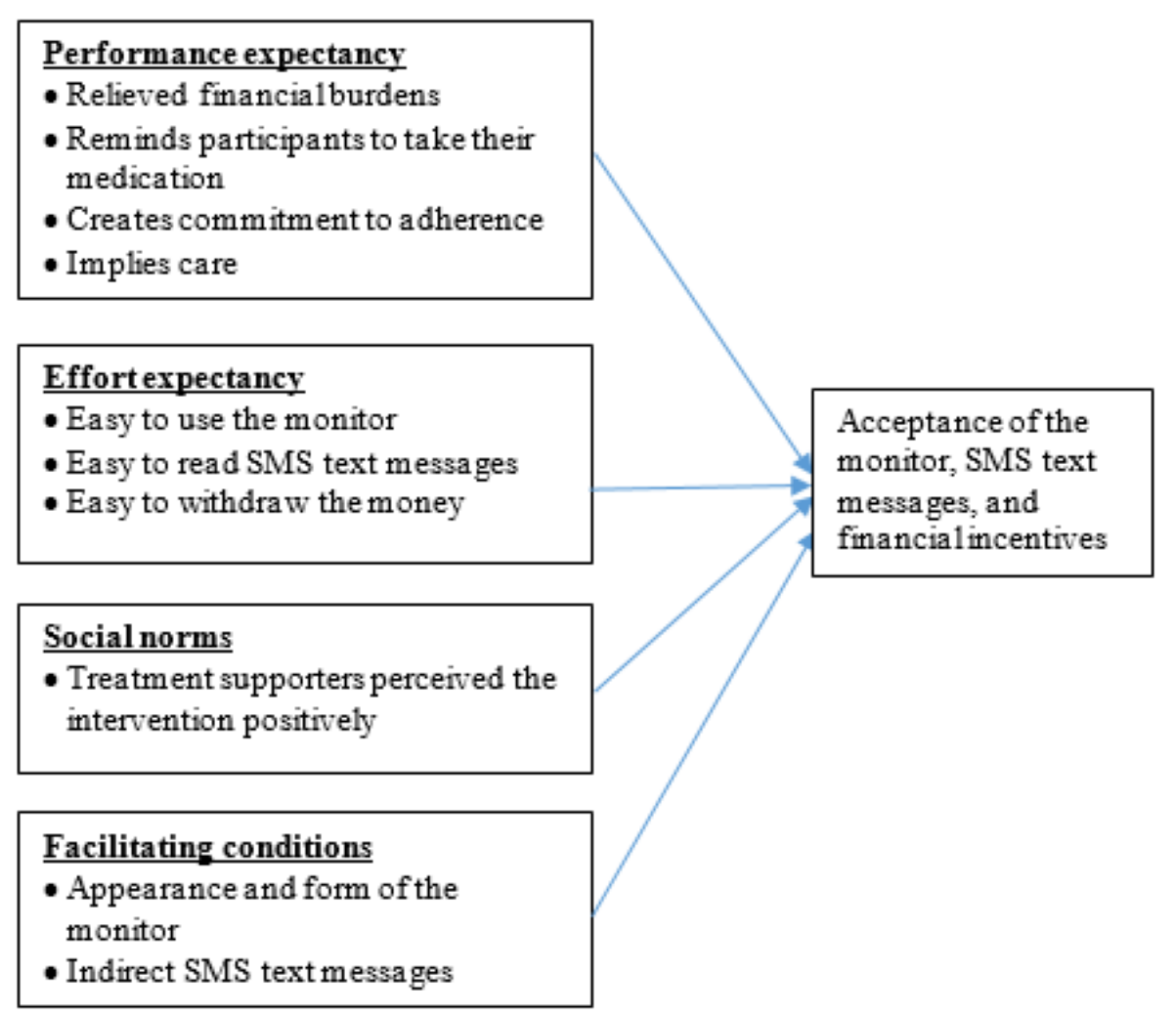
Organization of the qualitative data on acceptability following the unified theory of acceptance and use of technology (UTAUT) model.

Acceptability is presented following the constructs of the UTAUT model (Figure 3) of the performance expectancy, effort expectancy, social norms, and facilitating conditions associated with the intervention.

#### Performance Expectancy or Perceived Usefulness

##### Mobile Money Incentives Relieve Participants of Tuberculosis-Related Financial Burdens

Before being enrolled in the study, some of the participants struggled to meet the basic costs of their tuberculosis care, including transport to the tuberculosis clinic and the cost of food and drinks needed to take the medication. These were mainly participants with no regular source of income, as well as those staying far from the tuberculosis clinic. They described relying on begging and borrowing money to meet their tuberculosis treatment costs. Unfortunate instances where begging and attempts to borrow money were not successful resulted in missed clinic appointments for pill refills due to lack of transport, which consequently resulted in missed medication. Others reported not taking their medication due to lack of food and drinks because they feared becoming weaker after taking their medication on an empty stomach. Participants reported that the mobile money transport incentives enabled them to meet the cost of transport to the clinic (eg, for pill refills) as well as the costs of meeting the basic tuberculosis treatment needs, such as food and drinks for taking their medication, thereby relieving them (as their treatment supporters) of the financial burdens associated with tuberculosis treatment and care. One participant stated as follows:

Before you started sending me mobile money, there were times when I would request people to borrow me money or help me with transport to the hospital, but sometimes they would also not be in a position to give me money, so I would miss picking my medication from the hospital...I stopped working when I got sick. I stay far from the hospital.This study helped a lot by sending me [money for] transport to the hospital so that I don’t miss taking my medication on time.

Male patient, aged 61 years

Participants narrated how medication adherence incentives encouraged them to take their medication on time to meet the monthly target of ≥90% medication adherence, which in turn helped them meet the cost of the basic food and drinks they needed for taking medication:

I knew that I would be given money after getting ≥90% medication adherence, so I made sure that I was taking my medication well in order to be sent the money. This disease made me too weak to work; yet, I needed money to buy food and porridge.

Female patient, aged 51 years

Whenever I would receive a message about my adherence percentage, and it’s below, it would motivate me to be serious so that next time I don’t miss out again.

Male patient, aged 39 years

##### SMS Text Message Reminders and the Real-Time Adherence Monitor Enabled Participants to Take Their Medication on Time

Participants reported that the SMS text message reminders and monitor-based alarms enabled them to take their medication on time. These technologies served as medication reminders, thereby addressing forgetfulness, which was common in participants who were not yet used to taking medication regularly. They were also useful for busy participants who could easily forget their medication-taking time due to other competing demands on their time. Participants reported being able to make the necessary preparations (eg, obtaining food and drinks and going back home in case the medication had been left behind at home) after receiving the daily SMS text message reminders (which were sent 30 minutes before their medication-taking time), thereby enabling them to take their medication on time:

The SMS [text message] reminders were very helpful because I was still learning how to take medicine in time, so they helped me in getting used to medication taking because they were coming every day, so my body eventually got used to the time.

Female patient, aged 35 years

At times, I would get too busy at my video library and forget taking my medication, but whenever I would receive the message, I would close the business immediately, go home, eat some food, prepare a drink, and use it to take my medication on time.

Male patient, aged 27 years

#### Using a Real-Time Adherence Monitor Creates Commitment to Medication Adherence

Participants perceived real-time adherence monitoring as “being watched” while taking their medication. This perception was welcomed and encouraged them to take their medication on time to demonstrate their commitment to the health care providers who they felt were concerned about their health and would not be happy with nonadherence:

When I started using the device, I felt touched knowing that there are people who are concerned about my life to the extent of using the device to watch me take medication yet they are not even my relatives or friends or people I knew before. This gave me morale to swallow my medication to play my part especially because they would be seeing whether or not I am taking my medication and they would probably feel bad if I miss taking [it].

Female patient, aged 40 years

Whenever I felt like not taking the medication, I always got motivated me to take medication because I knew that you people cared for me so much to the extent that you gave me this monitor and kept texting me to remind me to take medication and even sent me money to go to the clinic. I felt encouraged because you were really interested in seeing my health condition improve.

Female patient, aged 26 years

Although monitoring created commitment to medication adherence, it is noteworthy that the primary motivation for taking medication on time as reported by participants was the need to recover their good health and live longer. A participant stated as follows:

Whenever I saw it [the device], I knew it was going to report me, so I chose to take the commitment of swallowing the medication. But, the main issue was, I really needed to recover from this disease because I loved my life and wanted to save it by getting well as soon as possible.

Male patient, aged 57 years

### Receiving Financial Incentives and Reminders and Being Monitored Implies Care

#### Overview

Receiving the mobile money incentives and SMS text message or alarm reminders and being monitored via the real-time adherence monitor was perceived by participants as signs that the health care providers cared about them, which eventually created emotional support and a sense of connectedness that countered depressive feelings. A participant describes how she changed her mind about committing suicide as a result of using the technologies:

I was about to stop taking the medication and die because I developed self-rejection. I was in pain, and I had no one to help me, but you people encouraged me to take the medication when you put me in this study and started sending me texts, alarms, and mobile money to support me to take my medication. I dropped the idea of suicide because you people cared for me and loved me even more than I loved myself. Thank you for saving my life because I would be dead by now.

Female patient, aged 33 years

#### Effort Expectancy or Perceived Ease of Use: The Intervention Was Easy to Use

After the participants’ initial orientation to using the real-time adherence monitor, they found it easy to use for taking their medication. Participants, including a few (3/40, 8%) who never went to school, reported finding it easy to read the SMS text messages sent to them. In addition, they reported that it was easy to withdraw money from mobile money agents because the agents are readily available:

It was very easy to use the container [the real-time adherence monitor]; you open it the same way a food box is [opened], put your medication [in], and start using it; that is all. I did not have to charge it or do any other thing with it.

Male patient, aged 42 years

Although I did not go to school, I can read messages written in my local language, so, reading the messages on [the mobile] phone was not a problem at all.

Female patient, aged 28 years

There are so many mobile money agents around. It was easy for me to withdraw my money from them.

Male patient, aged 35 years

#### Social Norms or Other People’s Perceptions of the Intervention

#### Positive Perceptions From Treatment Supporters

Participants reported that their treatment supporters approve of them using the intervention to support their medication adherence to get well. In addition, participants stated that because of the financial incentives, their treatment supporters were relieved of the financial burden of having to take care of the financial needs of the participants:

When my wife saw the container and the messages, she was happy knowing that I was being supported by the hospital; she believed the support would help [me] get well soon. It was also a relief for her when you sent me money; I stopped working when I got sick, and before your assistance, I was only relying on getting money from her small shop for transport to the hospital and getting other basic needs like food.

Male patient, aged 32 years

#### Possibility of Inappropriate Use of the Financial Incentives

One participant reported how her husband initially misappropriated the financial incentives intended to pay for her transport to the clinic. Although the participant did not miss visiting the clinic, she had to keep begging her husband for money for transport to the clinic. Sometimes she would have to walk part of the distance due to insufficient transport funds provided by her husband:

My husband never wants me to own any money and always forces [me] to give him my money. So, whenever you would send me money on the [mobile] phone, he would force me to give him the whole of it. I would then have to go through the hassle of begging him to give me the money for my hospital visit, and sometimes the money he would give me for transport would not be enough.... But after giving you my new SIM card [details], which he did not know [about], I started receiving and using the money for transport to the hospital.

Female patient, aged 33 years

### Facilitating Conditions: Appearance and Form of the Monitor

Participants reported that they liked the appearance and form of the real-time adherence monitor. Specifically, they liked the monitor’s design, which resembled a food box, and its size, which they thought was reasonable because it accommodated all their pills; the absence of tuberculosis-related labels that could link them to the disease; and the hard outer cover that kept their medicines safe and clean, all of which motivated them to use the monitor:

The container looks like a food bowl, so people can easily think you have carrying some food in it; it is also big enough to carry all my medicine, and has no any TB-related word.

Female patient, aged 35 years

### Indirect SMS Text Messages

To avoid unwanted status disclosure, participants preferred SMS text messages that could not easily link them to tuberculosis:

I chose the message “come and eat” because for me I knew what it reminded me to eat, but other people even if they saw it on my [mobile] phone would not know what I was going to eat.

Male patient, aged 38 years

## Discussion

### Principal Findings

Drawing on the UTAUT model, this paper describes the feasibility and acceptability of My Mobile Wallet, a DAT intervention composed of a real-time adherence monitor, SMS text message reminders, and mobile money incentives (WiseCash) for tuberculosis medication adherence in rural southwestern Uganda. Generally, we found that the intervention was technically feasible because it functioned as expected. All participants reported that it was easy for them to use the intervention; they could access and read the mobile money SMS text messages as well as the medication reminders, and they were able to open the Wisepill device to retrieve their medication. Participants reported receiving the mobile money incentives for transport to the clinic and the medication adherence incentives as expected. The SMS text messages and real-time adherence monitor also worked as expected: the SMS text messages were sent as planned, the transmission of adherence data from the monitor worked well, and no monitor malfunctioned for the entire period of the study.

Concerning acceptability, participants reported being relieved of tuberculosis-related financial burdens as a result of receiving the mobile money incentives. SMS text message reminders and real-time monitor-based alarms reminded participants to take their medication on time. Daily SMS text message reminders were preferred to reminders triggered by missed doses. Participants’ preference for daily SMS text message reminders even in the treatment continuation phase (from month 4 onward) was surprising because one would assume that during this phase, they were nearly getting used to taking their medication and therefore did not require to be reminded daily. Patients’ preference for daily SMS text message reminders (for taking their medications) over weekly SMS text message reminders was also reported in our previous tuberculosis study [4] and HIV study [25]. As tuberculosis medications are taken daily, daily SMS text message reminders are preferred because they are aligned with the medication-taking frequency.

Participants perceived real-time adherence monitoring as “being watched” while taking their medication, which was welcomed and encouraged them to take their medication on time to demonstrate their commitment. Receiving the mobile money incentives and SMS text message or alarm reminders and being monitored via the real-time adherence monitor was perceived by participants as signs that the health care providers cared about them. Their experiences with the intervention eventually created emotional support and a sense of connectedness that countered depressive feelings among the participants. Inappropriate use of the mobile money transport incentives was reported only rarely.

### Limitations

The main limitation of this study is the possibility of social desirability bias in the data collected from interviews and surveys. The mobile money incentives in particular may have influenced participants, given the prevalence of poverty-based structural barriers to tuberculosis treatment in Uganda, a low-resource setting.

### Comparison With Prior Work

We are not aware of any study that reports the impact of a DAT intervention composed of a real-time adherence monitor, SMS text message reminders, and mobile money incentives on tuberculosis medication adherence. However, it should be noted that some studies using some components of this intervention exist; for instance, a recent systematic review and meta-analysis on cash interventions to improve tuberculosis outcomes concluded that these interventions could improve tuberculosis treatment success and completion among patients in low- and middle-income countries [10]; in this review, only 1 randomized control trial in Peru [26] was identified, and the authors of the review noted that the evidence is still weak. In addition, the use of face-to-face cash transfers or transport vouchers as incentives has been reported to be acceptable in facilitating adherence to tuberculosis diagnostic evaluation in Uganda [27]. Furthermore, receiving monthly financial incentives face-to-face enabled patients living with tuberculosis in Nigeria to purchase food and get transport to the clinic [28], while, in Uganda, receiving a one-time cash transfer upon sputum submission supported tuberculosis testing completion among patients [29]. Although the receipt of unconditional cash transfers through a direct benefit scheme supported registered patients living with tuberculosis to meet their nutrition requirements in India, the scheme had no significant effect on treatment outcome [30]. In our study, individuals with no regular source of income and those living far from the clinic benefited most from the mobile money incentives; participants used the transport incentives to cover the cost of transport to the clinic, while they used the financial incentives conditional on high medication adherence to buy food and drinks required to take their medication. This approach could potentially address the financial insecurities that continue to constrain medication adherence [4]. In Uganda, 53% of the patients living with tuberculosis take loans or sell property to meet the costs of their tuberculosis care [8]. Although there is limited research in this area, our study indicates that mobile money incentives can potentially relieve the financial burden that tuberculosis places not only on patients but also on their treatment supporters. An incentive as small as US $1 can increase the tuberculosis cure rate and reduce treatment loss to follow-up in Uganda [31]. Although this study estimated an average transport cost of US $8 (based on the COVID-19–pandemic-induced transport cost increases) and provided the same amount for transport to all participants, using GPS information to estimate and provide transport costs according to each participant’s distance from their home to the clinic could be a better option.

The reported practice of a husband taking the mobile money transport incentive from the wife shows the effects of poverty as well as the complexity of implementing mobile money incentives in low-resource settings and cultures where some people still believe in male-exclusive ownership of resources [32]. This scenario could result in an inappropriate use of the incentives, thus limiting the impact of the intervention. An inclusive approach that engages men in the implementation of such an intervention (eg, through awareness creation) might mitigate the risk of the incentives being misappropriated.

This is the first study to report on the feasibility of real-time monitoring linked to SMS text message reminders and mobile money incentives for tuberculosis medication adherence. In the same setting, we had previously reported that using SMS text message reminders linked to real-time monitoring is feasible and acceptable for supporting tuberculosis medication adherence [6]. This study provides insights regarding the integration of financial incentives with these technologies to support access to tuberculosis medication from the hospital and motivate medication adherence.

Participants’ medication adherence ascertained from the real-time adherence monitor was quite high. The receipt of financial incentives that was conditional upon a particular adherence target (≥90%) resulted in participants taking their medication on time in order to hit the target for financial incentives. In addition, participants’ awareness of the fact that their medication adherence was being watched or monitored through the real-time monitor motivated them to take their medication well in order not to disappoint those monitoring them. Notably, the reported adherence was ascertained from the monitor in the form of the monitor being opened, which was used as a proxy for medication taking. Overall, the real-time monitoring approach can potentially be more reliable than participant self-reports, which are highly subject to social desirability bias. However, although it was not reported in our findings, instances of opening the monitor without taking medication (such as accidental openings or opening the monitor to increase the chances of getting incentives) may constrain the feasibility of the intervention. In the ongoing phase of the study, we are supplementing the real-time adherence monitoring with hair analysis (assessing tuberculosis drug levels in participants’ hair) to improve objectivity.

Although a few SMS text messages (184/7064, 2.6%) and some adherence data (94/5776, 1.63%) could not be sent by the SMS text messaging application and the real-time adherence monitor, respectively, mainly due to technical issues such as poor network, the intervention was otherwise feasible. The feasibility of this intervention could be attributed to the rapid evolution and adoption of mobile phone technologies in Uganda, including among populations based in rural areas and considered economically marginalized [33]. The applications for the SMS text message reminders and mobile money incentives were tailored from the existing mobile phone infrastructure, which likely facilitated use by participants who were already familiar using SMS text messaging and mobile money services in their regular routines. By leveraging the existing mobile phone infrastructure, these technologies can potentially bridge the current gaps in access to health care services between economically advantaged populations and populations considered disadvantaged, consequently contributing to equitable access to health care. In addition, the fact that all participants owned personal mobile phones, had the ability to read SMS text message reminders and mobile money SMS text messages, and had reliable mobile network (per the enrolment criteria) could have contributed to the feasibility of the intervention. Different feasibility results may be yielded if this intervention is implemented in populations with fewer resources or in privileged populations.

Concerning acceptability, participants perceived the intervention’s functionalities of sending timely medication-taking reminders (through SMS text messages and monitor-based alarms), financially supporting medication adherence (through mobile money incentives), and monitoring medication taking (through the adherence monitor) as supportive and taking care of them. For the participants, this perception created a sense of connectedness with health care providers and countered depressive feelings after their tuberculosis diagnosis. It also encouraged them to adhere to taking their medication as a way of appreciating the care and proving their commitment to taking an active role in their own health with the ultimate goal of regaining their health. Such emotional support can also potentially empower patients to cope with the stigma and discrimination that are often associated with tuberculosis [34]. Importantly, there is evidence that emotional and social support can improve tuberculosis treatment success rates [35]. The use of various tuberculosis medication adherence technologies (including SMS text messages and real-time adherence monitors) was perceived by participants to reduce visits to clinics and increase access to social supporters in a variety of settings [36]. In South Africa, the use of a real-time adherence monitor (Wisepill evriMED1000) was acceptable for prompting a stepwise differentiated care approach for tuberculosis medication adherence, composed of SMS text messages, telephone calls, home visits, and motivational counseling, in response to missed doses ascertained from the monitor [37]. Other studies referencing patient experiences of using real-time adherence monitoring linked to SMS text message reminders for antiretroviral adherence support among people living with HIV in Uganda also reported perceptions of being cared for as a result of using the technologies [25]. Although there are differences between HIV and tuberculosis, they are both diseases of poverty, and result in stigma, and discrimination. The findings regarding the adherence monitor and the SMS text message aspects of the intervention were indeed similar in this and another [25] study, indicating the strong potential of the intervention in this and potentially other similar settings.

### Conclusions

In sum, we found the My Mobile Wallet intervention (composed of real-time adherence monitoring linked to SMS text message reminders and mobile money incentives) for tuberculosis medication adherence to be feasible and acceptable in a low-resource setting where poverty-based structural barriers heavily constrain tuberculosis treatment and care. The intervention worked as expected, and participants found it easy to use. The intervention relieved participants of the burden of tuberculosis treatment costs, reminded them to take their medication on time, and provided emotional support that made them feel connected to care.

On the basis of the findings from this study, we are now planning a randomized controlled trial (registered on ClinicalTrials.gov; NCT05656287) for assessing the full-scale feasibility, acceptability, and impact of My Mobile Wallet.

## Abbreviations

COREQ: Consolidated Criteria for Reporting Qualitative Research
DAT: digital adherence technology
MRRH: Mbarara Regional Referral Hospital
UTAUT: unified theory of acceptance and use of technology

## Data Availability

The data sets generated and analyzed during this study are available from the corresponding author on reasonable request.
